# The final cut: a multi-centre cohort study evaluating outcomes of robotic completion cholecystectomy

**DOI:** 10.1007/s00464-025-12044-1

**Published:** 2025-08-28

**Authors:** Irena Stefanova, Rosie Callahan, Vandana B. Giriradder, Nabeel Merali, Lydia Renardson, Shi Lam, Siong-Seng Liau, Esther Platt, Angela Riga, Rajesh Kumar, Tim R. Worthington, Adam E. Frampton, Rajiv P. Lahiri, Tim D. Pencavel, Jawad Ahmad

**Affiliations:** 1 Royal Liverpool University Hospital NHS Trust, Liverpool, UK; 2 HPB Surgical Unit, Royal Surrey Hospital NHS Trust, Guildford, UK; 3 University Hospitals Coventry and Warwickshire NHS Trust, Coventry, UK; 4 Cambridge University Hospitals NHS Trust, Cambridge, UK; 5 Section of Oncology, FHMS, University of Surrey, Guildford, UK

**Keywords:** Completion cholecystectomy, Robotic surgery, Subtotal cholecystectomy, Abandoned cholecystectomy, Remnant gallbladder

## Abstract

**Background:**

Subtotal and abandoned cholecystectomies are on the rise due to the increase of laparoscopic cholecystectomies performed in the emergency setting. Persistent biliary symptoms postoperatively may necessitate a completion cholecystectomy (CC) which is a technically challenging procedure. The literature describing outcomes of minimally invasive CC is scarce and consisting of small studies only. This retrospective multi-centre study aimed to assess the safety and effectiveness of the robotic approach to CC.

**Methods:**

All consecutive patients (> 18 years), undergoing a robotic CC between August 2020 and March 2025, were included. Primary outcome was success of robotic procedure. Secondary outcomes were open conversion, subtotal and abandoned cholecystectomy rate, postoperative complications, length of hospital stay, 30-day re-admission and 90-day mortality. A *P* value < 0.05 was considered significant. Data were analysed using IBM SPSS Statistics Version 29.0.1.0 (171).

**Results:**

A total of 44 adult patients with a median age was 58.5 years (IQR: 43.5 – 73.3) undergoing robotic CC were included. Previous subtotal cholecystectomy was performed in 72.7% (32/44) of referrals, whereas abandoned cholecystectomy was recorded in 27.3% (12/44).

There were no abandoned robotic CCs. Subtotal cholecystectomy was required in two cases (4.5%, 2/44), and conversion to open procedure was reported in one case 2.3% (1/44). A successful robotic CC was documented in 93.2% (41/44) of procedures. Overall morbidity rate was 6.8% (3/44) with a Clavien-Dindo grade ≥ 3 in 2.3% (1/44) of cases. All complications were observed in the previous subtotal cholecystectomy group (9.4%, 3/32) vs the abandoned cholecystectomy group (0%, 0/12), however, this difference was not significant (*P* = 0.55, 95% CI [-0.065, 0.25]).

**Conclusion:**

Robotic CC is a safe and technically feasible approach with high success and low complication rates. This study, the largest of its kind, supports the expanding role of robotic platforms in managing difficult gallbladder pathology in the re-operative setting.

Cholecystectomy remains one of the most frequently performed general surgical procedures in the United Kingdom, with laparoscopic cholecystectomy considered the gold standard management of symptomatic gallstone disease [1]. The current recommendations are to perform laparoscopic cholecystectomy during the index emergency admission with acute gallstone pathology such as severe acute cholecystitis, cholangitis or biliary pancreatitis [2]. As a result, surgeons are faced with increasingly “difficult gallbladders” associated with an increased risk of intraoperative complications.

During the evolution of laparoscopic cholecystectomy, the identification of ‘critical view of safety’ (CVS) was widely advocated for, in the effort to reduce the rates of bile duct injury [3–5]. However, in cases of severe inflammation anatomy can be distorted precluding a clear recognition of CVS. In procedures with high risk of injury to critical structures, performing a subtotal cholecystectomy is favoured [6, 7]. Consequently, the rate of subtotal cholecystectomy is increasing [1, 8]. Nonetheless, the presence of a gallbladder remnant after subtotal cholecystectomy can lead to persistent symptoms and hospital re-admissions. The incidence of recurrent biliary symptoms varies between 9 and 18% and can necessitate performing a completion cholecystectomy (CC) which is a technically challenging procedure [9].

Traditionally CC was performed via an open approach, however, the benefits of minimally invasive surgery (MIS) have made laparoscopic CC more popular [10, 11]. Furthermore, the robotic platforms are now increasingly utilised in complex hepatobiliary procedures including CC [12]. The improved vision, dexterity and intraoperative adjuncts, such as Indocyanine Green (ICG), have clear benefits over standard laparoscopic approaches in a hostile re-operative field [12, 13].

The literature reporting on both laparoscopic and robotic CC outcomes is still scarce consisting of small studies only [13–15]. Without robust data it is difficult to ascertain whether these procedures are consistently safe and effective. Larger studies are required to investigate predictors of success or failure.

This multi-site, retrospective, cohort study aimed to evaluate the role of robotic CC following a previous subtotal cholecystectomy or abandoned cholecystectomy. To our knowledge, this is the largest series reporting the outcomes of robotic CC to date.

## Methods

### Study design and ethics considerations

This UK-based multi-centre retrospective observational study reported data from three tertiary hepato-pancreato-biliary (HPB) centres. The STROBE reporting criteria for cohort studies were followed. Considering the retrospective nature of this study utilising only fully anonymised data for analysis, ethical approval was not sought.

### Patient selection

The inclusion criteria comprised of all consecutive adult patients (above the age of 18 years) who underwent robotic CC in the respective centres with an indication of either a previous subtotal cholecystectomy or abandoned cholecystectomy elsewhere. The procedures were performed between August 2020 and March 2025. This period included all robotic CC cases from the initiation of robotic biliary surgery in each unit to the study end date.

### Procedure

Robotic CC was performed using the Da Vinci XI or X platform (Intuitive Surgical Sunnyvale CA). Patient position was supine with 15 degrees of reverse Trendelenburg. Pneumoperitoneum was induced using the open Hassan technique via a 12 mm infraumbilical assistant port. Four 8 mm robotic ports were introduced in the upper abdomen 8 cm apart. The use of ICG, Intraoperative Cholangiogram (IOC), Intraoperative Ultrasound (IOUS), or choledochoscopy was on the discretion of the operating surgeon and on case-by-case basis.

### Study outcomes

The primary outcome was success in performing a CC robotically. Secondary outcomes included conversion to open procedure, subtotal cholecystectomy rate, abandoned procedure rate, postoperative complications and interventions rate, length of hospital stay (LOS), 30-day re-admission rate and 90-day mortality rate.

### Data collection

Retrospective data collection using electronic hospital records was carried out by each centre independently. Subsequently, the data were fully anonymised prior to submitting for analysis. The acquired data included: age, gender, Body Mass Index (BMI), American Society of Anaesthesiologists (ASA) grade, Charlson Comorbidities Index (CCI), past surgical history, tertiary centre referral reason, urgency of procedure, intraoperative findings, operative time, and outcomes. Postoperative complications were graded using the Clavien-Dindo classification [16]. All patients underwent a telephone follow-up between 3 and 6 months postoperatively to assess for recurrence of biliary symptoms. In addition, medical records were reviewed during data collection to identify any subsequent hospital admissions related to biliary disease.

### Statistical analysis

Continuous variables were reported as median and interquartile range (IQR), while categorical data were presented as numbers and percentages. Fisher’s exact test was utilised to determine association between binary categorical variables. A *P* value < 0.05 was considered significant. Data were analysed using IBM SPSS Statistics Version 29.0.1.0 (171).

## Results

A total of 44 consecutive patients underwent a robotic CC during the study period.

### Baseline demographic characteristics

The majority were female (63%, 28/44). The median age was 58.5 years (IQR: 43.5 – 73.3), with a median BMI of 35 kg/m^2^ (IQR: 29.7 – 38.1). Patients were predominantly categorised as ASA grade 2 (61.4%, 27/44) or ASA grade 3 (29.6%, 13/44). The median CCI was 1.9 (IQR: 0 – 3) (Table 1).

**Table 1:** Baseline demographic characteristics

		**Total number (%)**
Age (median)	58.5 years (IQR: 43.5 – 73.3)	
Gender	Female	28/44 (63.6%)
	Male	16/44 (36.4%)
BMI (median)	35 kg/m^2^(IQR: 29.7 – 38.1)	
CCI	0	12/44 (27.3%)
	1	9/44 (20.5%)
	2	7/44 (15.9%)
	3	8/44 (18.2%)
	4	4/44 (9.1%)
	5	4/44 (9.1%)
ASA grade	1	3/44 (6.8%)
	2	27/44 (61.4%)
	3	13/44 (29.6%)
	4	1/44 (2.3%)
Medical comorbidities	Hypertension	10/44 (22.7%)
	Type 2 diabetes mellitus	9/44 (20.5%)
	Hypothyroidism	7/44 (15.9%)
	Pulmonary embolism	4/44 (9.1%)
	Atrial fibrillation	4/44 (9.1%)
	Transient ischaemic attack	3/44 (6.8%)
	Deep vein thrombosis	3/44 (6.8%)
	COPD	3/44 (6.8%)
	Ischaemic heart disease	3/44 (6.8%)
	Depression	3/44 (6.8%)
	CKD	1/44 (2.3%)
	Obstructive sleep apnoea	1/44 (2.3%)
	Asthma	1/44 (2.3%)
Past surgical history	Subtotal cholecystectomy	32/44 (72.7%)
	Abandoned cholecystectomy	12/44 (27.3%)

*IQR* Interquartile range; *BMI* Body mass index; *CCI* Charlson Comorbidities Index; *ASA* American Association of Anaesthesiologists; *COPD* Chronic obstructive pulmonary disease; *CKD* Chronic kidney disease

### Pre-operative details

Previous subtotal cholecystectomy was the referral reason for consideration of robotic CC by a tertiary HPB centre, in 72.7% (32/44) of the cases, whereas previous abandoned cholecystectomy was recorded in 27.3% (12/44) of the referrals.

In almost half of the cases (47.7%, 21/44), the reason to perform subtotal or abandoned cholecystectomy at the index operation was unknown. In 25%, severe cholecystitis precluded successfully performing a total cholecystectomy at first attempt. Other recorded reasons were adhesions (4.5%, 2/44), fistula (4.5%, 2/44), intraoperative bile leak (4.5%, 2/44), empyema (2.3%, 1/44), residual inflammation from previous severe pancreatitis (2.3%, 1/44). In 9.1% (4/44) the initial operation reported that a total cholecystectomy was carried out, however a remnant gallbladder was identified on postoperative Magnetic Resonance CholangioPancreatography (MRCP) after patients represented with biliary symptoms.

Prior to robotic procedure, 20.5% (9/44) of patients underwent an Endoscopic Retrograde CholangioPancreatography (ERCP) for the treatment of either choledocholithiasis or bile leak; one patient had a cholecystostomy for gallbladder empyema; and another patient underwent a Percutaneous Transhepatic Cholangiography (PTC). The median time from index operation to robotic CC was 11.5 months (IQR: 6.1 – 33.7) (Table 2).

**Table 2:** Pre-operative data

Referral reason	Previous subtotal cholecystectomy	32/44 (72.7%)
	Previous abandoned cholecystectomy	12/44 (27.3%)
Reason to perform subtotal cholecystectomy or abandon	Severe cholecystitis	11/44 (25%)
	Adhesions	2/44 (4.5%)
	Fistula	2/44 (4.5%)
	Empyema	1/44 (2.3%)
	Intraoperative Bile leak	2/44 (4.5%)
	Previous severe pancreatitis	1/44 (2.3%)
	Unknown	21/44 (47.7%)
	Index operation reported as total cholecystectomy	4/44 (9.1%)
Prior IR or endoscopy procedures	ERCP	9/44 (20.5%)
	Cholecystostomy	1/44 (2.3%)
	PTC	1/44 (2.3%)
Time from original operation to completion cholecystectomy (median)	11.5 months (IQR: 6.1 – 33.7)	

*ERCP* Endoscopic retrograde cholangiopancreatography; *PTC* Percutaneous transhepatic cholangiography; *IR* Interventional radiology; *IQR* Interquartile range

### Technical considerations

All procedures were undertaken as elective cases. ICG was the preferred method of intraoperative visualisation of the biliary tree in 25% (11/44) of patients, whereas IOUS was used in 4.5% (2/44), a combination of ICG and IOUS in 4.5% (2/44), IOC in 2.3% (1/44) and ICG alongside choledochoscopy in 2.3% (1/44) of cases.

The most common intraoperative findings were dense adhesions in 47.7% (21/44), followed by a type 1 Mirizzi syndrome (11.4%, 5/44), a cholecystoduodenal fistula (6.8%, 3/44), a cholecystocolonic fistula (4.5%, 2/44), and a cholecystocutaneous fistula (4.5%, 2/44).

Critical view of safety was documented as ‘seen’ in 52.3% (23/44) of robotic CCs. The most common method of securing the cystic duct was using Hem-o-lok clips (56.8%, 25/44), however 3/0 V-lok (13.6%, 6/44), 3/0 PDS (2.3%, 1/44), 4/0 PDS 2.3%, 1/44) and 5/0 PDS 2.3%, 1/44) and 3/0 Stratafix 2.3%, 1/44), were also utilised for closure of the cystic duct stump or the side of bile duct. Intra-abdominal drains were placed in 47.7% (21/44) of procedures.

There were no abandoned robotic CCs. Subtotal cholecystectomy was required in two cases (4.5%, 2/44), whereas conversion to open procedure was reported in one case 2.3% (1/44). In all three cases, dense adhesions and a frozen Calot’s triangle were encountered, precluding safe dissection. Additionally, one of the subtotal procedures revealed concurrent cholecystoduodenal and cholecystocolonic fistulae, further complicating the operative field. A successful robotic CC was documented in 93.2% (41/44) of procedures (Table 3).

**Table 3:** Robotic completion cholecystectomy—Procedure details

Procedure urgency	Elective	44/44 (100%)
	Emergency	0/44 (0%)
Intraoperative visualisation of biliary tree	ICG	11/44 (25%)
	IOUS	2/44 (4.5%)
	ICG + IOUS	2/44 (4.5%)
	IOC	1/44 (2.3%)
	ICG + Choledochoscopy	1/44 (2.3%)
Intraoperative findings	Dense adhesions	21/44 (47.7%)
	Dense adhesions and chronic abscess	2/44 (4.5%)
	Free gallstones and chronic abscess	1/44 (2.3%)
	Cholecystoduodenal fistula	3/44 (6.8%)
	Cholecystocolonic fistula	2/44 (4.5%)
	Cholecystocutaneous fistula	2/44 (4.5%)
	Combination of cholecystoduodenal and cholecystocolonic fistulae	1/44 (2.3%)
	Type 1 Mirizzi	5/44 (11.4%)
	Type 2 Mirizzi	1/44 (2.3%)
	Unknown	6/44 (13.6%)
CVS documented as seen	Yes	23/44 (52.3%)
	No	21/44 (47.7%)
Management of cystic duct/side of CBD	Hem-o-lok clip	25/44 (56.8%)
	Continuous 3/0 V-Lok	6/44 (13.6%)
	Interrupted 3/0 PDS	1/44 (2.3%)
	Interrupted 4/0 PDS	1/44 (2.3%)
	Interrupted 5/0 PDS	1/44 (2.3%)
	Continuous 3/0 Stratafix	1/44 (2.3%)
	Unknown	9/44 (20.5%)
Intra-abdominal drain placement	Yes	21/44 (47.7%)
	No	23/44 (52.3%)
Open conversion	Yes	1/44 (2.3%)
	No	43/44 (97.7%)
Abandoned procedure	Yes	0/44 (0%)
	No	44/44 (100%)
Subtotal cholecystectomy	Yes	2/44 (4.5%)
	No	42/44 (95.5%)
Successful robotic completion cholecystectomy	Yes	41/44 (93.2%)
	No (incl. 1 open conversion and 2 subtotal procedures)	3/44 (6.8%)
Operative time (median)	112 min (IQR: 83.5 – 140.5)	

*ICG* Indocyanine green; *IOUS* Intraoperative ultrasound; *IOC* Intraoperative cholangiogram; *CVS* Critical view of safety; *CBD* Common bile duct; *IQR* Interquartile range

### Postoperative outcomes

The overall morbidity rate was 6.8% (3/44). The main complications included: one patient (2.3%, 1/44) with a retained common bile duct stone requiring a postoperative ERCP; one patient (2.3%, 1/44) with a perihepatic collection which was treated with an antibiotic course; and another individual who had a wound infection (2.3%, 1/44) (Table 4). All observed complications were in the previous subtotal cholecystectomy group (9.4%, 3/32) vs the previously abandoned cholecystectomy group (0%, 0/12); however, this difference was not statistically significant (Fisher’s exact, 2-sided, *P* = 0.55, 95% CI [-0.065, 0.25] with continuity correction). There were no patients requiring a return to theatre or a postoperative interventional radiology procedure. The highest grade of Clavien-Dindo complications was 3a (2.3%, 1/44).

**Table 4:** Postoperative complications

Complications	Retained CBD stone	1/44 (2.3%)
	Perihepatic collection	1/44 (2.3%)
	Wound infection	1/44 (2.3%)
Clavien – Dindo class	1	0/44 (0%)
	2	2/44 (4.5%)
	3a	1/44 (2.3%)
	3b	0/44 (0%)
	4	0/44 (0%)
	5	0/44 (0%)
Postoperative interventions	ERCP	1/44 (2.3%)
Overall morbidity		3/44 (6.8%)
Length of stay (median, days)		1 (IQR: 0 – 2)
30 – day re-admission		6/44 (13.6%)
90 – day mortality		0/44 (0%)

*CBD* Common bile duct; *ERCP* Endoscopic retrograde cholangiopancreatography; *IQR* Interquartile range

The median LOS was 1 day (IQR: 0 – 2). The 30-day re-admission rate was 13.6% (6/44). Three patients (6.8%, 3/44) represented with postoperative pain; however, their laboratory blood tests, and postoperative Computed Tomography (CT) scans did not identify any abnormalities. These individuals were therefore discharged from hospital with analgesics. There were no recorded 90-day mortalities (Table 4).

## Discussion

This is the largest study to date demonstrating that robotic completion cholecystectomy is technically feasible and safe, associated with high success rates of 93.2%, and low rates of open conversion (2.3%), even in the challenging setting of re-operative biliary surgery.

Most studies on robotic completion cholecystectomy consist of case series, or case reports, with only one study reporting 26 cases. Tschuor et al. did not observe any conversions to open procedure in their cohort, and reported 11.5% overall morbidity rate [15]. Our results were similar, with one open conversion, but a lower morbidity rate of 6.8%, with only one grade 3a complication. In comparison, the literature is more inconsistent in describing the outcomes of laparoscopic completion cholecystectomy. A recent study by Zhu et al*.* revealed a low open conversion rate of 4.4% in 46 laparoscopic cases, with overall morbidity of 19.6%, 8.7% of which were major complications [17]. Whereas, another study, including 48 patients, demonstrated a conversion rate as high as 20.4%, with 11% morbidity rate following a laparoscopic completion cholecystectomy [18]. These latter findings were reaffirmed by Etherington et al. who reported that out of 68 completion cholecystectomies attempted laparoscopically, 22 were converted to laparotomy (32.4%) [8]. The laparoscopic approach to completion cholecystectomy is well-established, however limitations related to rigidity of instruments with fewer degrees of freedom, suboptimal visualisation and ergonomic strain, still remain, and are more noticeable in a re-operative field.

Beyond outcome comparisons, re-do biliary surgery is technically challenging both due to the original pathology necessitating a subtotal cholecystectomy, creating a hostile right upper quadrant, and the added complications resulting of recurrent pathology due to the remnant gallbladder. In our series of robotic completion cholecystectomy, 36 out of 44 patients (81.8%) had complex anatomical and pathological findings including 8 cases (18.2%) of cholecystoduodenal, cholecystocolonic and cholecystocutaneous fistula and 6 cases of Mirizzi syndrome. The fistula rate observed here was notably higher in comparison to Zhu et al*.* and Singh et *al*. studies, 8.7% and 9.7%, respectively. [17, 18] However, our postoperative outcomes were not influenced by this, and were not inferior to laparoscopic studies. Furthermore, there is increasing evidence of the successful application of robotic surgery in challenging gallstone pathology such as completion cholecystectomy, following complicated open subtotal cholecystectomies, or cholecystectomy in ‘difficult gallbladders’ [12, 14, 19]. In addition, Magge et al. presented 6 cases of preoperatively diagnosed Mirizzi syndrome which was managed in a single stage robotic procedure, with 3 procedures requiring biliary reconstruction with hepaticojejunostomy [20]. Although laparoscopic surgery is already widely incorporated in routine biliary surgery, and less costly, the growing evidence suggests that robotic platforms could be advantageous, in enhancing dexterity and improving vision, allowing fine dissection around critical structures in the re-operative field, and avoiding the need to convert to an open procedure.

In addition, robotic platforms facilitate the use of adjuncts such as near-infrared fluorescent cholangiography with ICG. In our series, ICG was employed in 31.8% of cases to aid in delineation of extrahepatic biliary anatomy. This technique has been shown to be faster and more cost-effective than intraoperative cholangiography (IOC) in laparoscopic surgery, with high rates of cystic duct visualization [21, 22]. Tschuor et al*.* also reported the use of ICG as an adjunct to intraoperative ultrasound in robotic cases, underscoring its growing role in complex biliary procedures potentially facilitating safer dissection [15].

## Study limitations

There are several limitations to this multi-centre study including its retrospective nature and resulting bias. A laparoscopic or open comparator group was not included due to the absence of reliable procedural data and standardised coding across participating institutions, which precluded retrospective identification and analysis of such cases. Nonetheless, the outcomes of the current study were interpreted in the context of the best available literature.

A formal cost–benefit analysis was beyond the scope of this investigation. However, given the current health economic climate, we acknowledge the critical importance of cost effectiveness and prudent resource allocation. Future studies should aim to address this aspect.

Finally, we recognise the limitations associated with small sample sizes, particularly in the subgroup analysis of complication rates. This impacts the statistical power to detect significant differences and introduces the risk of Type II error. Nevertheless, this represents the largest study to date reporting outcomes of robotic completion cholecystectomy. Larger cohorts are necessary to robustly identify predictors of procedure success or failure, and to contextualise outcomes within the framework of surgeons’ robotic learning curves.

## Conclusion

Robotic completion cholecystectomy is a safe and technically feasible approach in re-operative biliary surgery, with high success and low complication rates. This study, the largest of its kind, supports the expanding role of robotic platforms in managing difficult gallbladder pathology in the re-operative setting. Further research is needed to strengthen the evidence base.
